# Factors associated with long COVID syndrome in a Colombian cohort

**DOI:** 10.3389/fmed.2023.1325616

**Published:** 2023-12-22

**Authors:** María Camila Martínez-Ayala, Nadia Juliana Proaños, Julian Cala-Duran, Alvaro Jose Lora-Mantilla, Catalina Cáceres-Ramírez, Silvia Juliana Villabona-Flórez, Paul Anthony Camacho-López

**Affiliations:** ^1^School of Medicine, Universidad de La Sabana, Chía, Colombia; ^2^Directorate of Research, Development and Technological Innovation, Ophthalmological Foundation of Santander (FOSCAL), Floridablanca, Colombia

**Keywords:** COVID-19, SARS-CoV-2, post COVID condition, post-acute sequelae of COVID-19 (PASC), risk factors

## Abstract

**Introduction:**

After acute phase of SARS-CoV-2 infection, some patients persist with clinical symptoms, a phenomenon known as Long COVID syndrome. It is necessary to understand the factors associated with the persistence of these symptoms to develop individualized preventive approaches and effectively address this challenge.

**Objective:**

To determine the factors associated with the persistence of symptoms six months after COVID-19 infection.

**Materials and methods:**

A ambidirectional cohort, single-center study, that included individuals previously diagnosed with COVID-19 by real-time polymerase chain reaction (PCR) positive test, who were followed for a period of six months. Univariate, bivariate and a multivariate binomial regression model were performed to determine risk factors associated with the persistence of COVID-19 symptoms at the six months of follow-up.

**Results:**

The prevalence of long COVID syndrome was 47%. Age demonstrated no significant association with Long COVID (RR 0.999 [95% CI 0.996–1.002]); however, female sex (RR 1.148 [95% CI 1.038–1.268]), requirement of mechanical ventilation (RR 1.278 [95% CI 1.050–1.555]), presence of Chronic Obstructive Pulmonary Disease (COPD) (RR 1.340 [95% CI 1.104–1.626]), Rheumatic Disease (RR 1.259 [95% CI 1.055–1.504]) and the Hospitalization Type: General Hospitalization (RR 1.247 [95% CI 1.090–1.427]) and ICU Hospitalization (RR 1.490 [95% CI 1.221–1.818]) were significantly associated with the persistence of symptoms at the six month of follow-up.

**Conclusion:**

Female sex, presence of COPD, rheumatic disease, hospitalization type and requirement of mechanical ventilation during index infection were identified as significant risk factors for the diagnosis of Long COVID. These findings emphasize the importance of addressing Long COVID syndrome in terms of prevention and management, taking these risk factors into consideration.

## 1 Introduction

The COVID-19 pandemic, sparked by the emergence of the novel coronavirus, SARS-CoV-2, in late 2019, has had a profound global impact. It quickly spread worldwide, resulting in millions of infections and substantial mortality (1). While significant attention has been focused on managing and preventing the disease in its acute phase, there is growing concern about persistent health issues that linger long after the initial infection has subsided (2).

Long COVID (LC), also known as post-COVID conditions (PCC) or post-acute sequelae of COVID (PASC), is characterized by the persistence or recurrence of at least one symptom following an initial SARS-CoV-2 infection, as defined by the Center for Disease Control and Prevention (CDC) and the World Health Organization (WHO) (3, 4). Typically emerging around 3 months after the onset of COVID-19 symptoms, these symptoms endure for a minimum of two months. They may either arise as *de novo* occurrences after the primary convalescence or persist continuously from the initial illness, exhibiting temporal fluctuations or experiencing relapses over time (3, 4). Remarkably, these symptoms closely resemble those encountered during the acute phase of the disease (5), significantly impacting the quality of life and posing a substantial public health concern (2, 3).

As the pandemic unfolded, the primary focus was on immediate containment, diagnosis, and acute care, with limited consideration for the medium and long-term consequences of COVID-19 (6, 7). LC presents a significant challenge to public health and healthcare systems, not only due to its debilitating nature, but also because it affects a substantial proportion of individuals who have recovered from the initial infection (8, 9). It also carries economic implications, with significant direct medical costs and potential productivity losses (8, 10).

The long-term effects of COVID-19 infection on the health of survivors are still being investigated. Several studies have identified the persistence of LC up to one year after the illness, with the most prevalent symptoms during this period being fatigue/weakness, dyspnea, arthralgia, depression, and anxiety (11). A study conducted in Mexico revealed that over 83.3% of patients exhibited post-COVID conditions within 6 months of discharge (12). The study also found that the most common symptoms were headache, anosmia, ageusia, and cough, and that the severity of the initial illness was associated with the risk of developing post-COVID conditions (12).

Despite the increasing recognition of Long COVID (LC), there is still limited information available about the factors influencing its occurrence and severity. Existing reports on LC have offered valuable insights into its prevalence and symptomatology; however, persistent limitations and knowledge gaps persist. Understanding the underlying mechanisms of this condition, as well as the associated clinical and demographic characteristics, is crucial for developing targeted interventions and improving the quality of life for those affected (13). These factors are still not well understood, especially in tropical countries like Colombia. Moreover, the economic and public health implications of LC underscore the need for further research to guide evidence-based decision-making (14).

This study aims to identify the factors associated with Post-COVID Syndrome six months after SARS-CoV-2 infection, to contribute to a deeper understanding of LC and provide valuable insights into the factors associated with its persistence.

## 2 Materials and methods

An ambidirectional cohort, single-center study, was performed in a third-level reference hospital in Santander, Colombia. All patients with COVID-19 polymerase chain reaction (PCR) positive tests discharged from the institution between March 29, 2020, and September 27, 2021, were included. Patients were excluded if they could not be reached for follow-up at six months and patients suffering from pneumonia or acute respiratory distress syndrome (ARDS) due to causes unrelated to COVID-19.

### 2.1 Procedures

#### 2.1.1 Initial stage

Trained research personnel undertook data collection in the initial stage. Patient information, including demographics, was sourced from their medical records, and telephone interviews were conducted for data validation and completion. The data collection process utilized LimeSurvey, an electronic survey software, to minimize missing entries and enable real-time data validation (15). Information was gathered from diagnosis, including in patients who did not require hospitalization. Baseline characteristics, such as demographic details, comorbidities (coded through International Classification of Diseases, 10th Revision [ICD-10] diagnosis codes), outpatient medication (including self-administered therapies for COVID-19 prevention or symptom management), smoking status, acute COVID-19 symptoms, and physical signs at emergency room (ER) admission, were documented. The baseline characteristics of the cohort had been previously published (16).

#### 2.1.2 Follow-up

Six months after the initial PCR COVID-19 positive test, patients underwent telephone interviews. Multiple attempts on different dates were made to contact patients or their families, and exclusion occurred if there was no response after five attempts. The follow-up process comprised four sections: identification, self-reported remaining symptoms, new comorbidities, or medications initiated post-hospital discharge, and laboratories/images. After completing identification information, any remaining COVID-19 symptoms were assessed, including rhinorrhea, myalgia, ageusia, anosmia, fatigue, etc. The course (since the acute disease or new onset) and severity of any present symptom were evaluated. Additionally, information on COVID-19 reinfection, ER consultations, or hospitalizations was gathered. Subsequently, information on newly diagnosed medical conditions by healthcare personnel, documented via ICD-10 diagnosis codes was collected.

### 2.2 Variables

The assessed variables in this study encompass several facets, including demographic characteristics such as age, sex, level of education, occupation, and socioeconomic status; clinical factors such as comorbidities, smoking history, exposure to biomass combustion; COVID-19 symptoms in the index contact and COVID-19-related care in the index contact. Further data collected at the six-month follow-up includes persistence and evolution of symptoms, newly diagnosed pathologies, and other pertinent information. LC was defined as the presence of at least one persistent or recurrent symptom six months after the infection (4).

### 2.3 Sample size

We estimated that the study would require a minimum sample size of 240 patients, in the more demanding scenario of 50% of patients diagnosed with LC. These patients would be sufficient to build a binomial regression multivariate model with 8 covariates (10 patients with the outcome per covariate) (17).

### 2.4 Statistical analysis

The normality of continuous variables was assessed using the Shapiro–Wilk test, showing a non-normal distribution in all of them. Data for continuous variables are presented as medians with interquartile ranges (IQR), and for categorical variables as absolute values with percentages. The Mann–Whitney U test was applied for the LC outcome in continuous variables, and chi-square for categorical variables, and crude risk ratios were calculated.

A multivariate binomial regression with log link function was performed to determine the variables associated with the long covid, variables with *p* value (< 0.25) in the initial bivariate analysis and biological plausibility were included in the multivariate model concerning the outcome of symptom persistence (17). The model was evaluated in terms of the AUC- ROC curve.

All hypothesis tests adhere to a two-tailed approach, with statistical significance defined as *p* < 0.05. For the analysis the Stata 17 statistical program was used (StataCorp., 2017, College Station, Texas: StataCorp LLC).

### 2.5 Ethical considerations

The study received ethical approval from the Fundación Oftalmologica de Santander Ethics and Research Committee” (Acta N° 624 22/09/2023) and The Research Subcommittee of the Universidad de la Sabana (Reference: MEDMSc-89-2023) considering it as risk-free research according to resolution 8,430 of 1993 and was conducted in accord with the principles of the Declaration of Helsinki. All participants gave written informed consent for data collection, analysis, and record linkage.

## 3 Results

**Figure 1:**
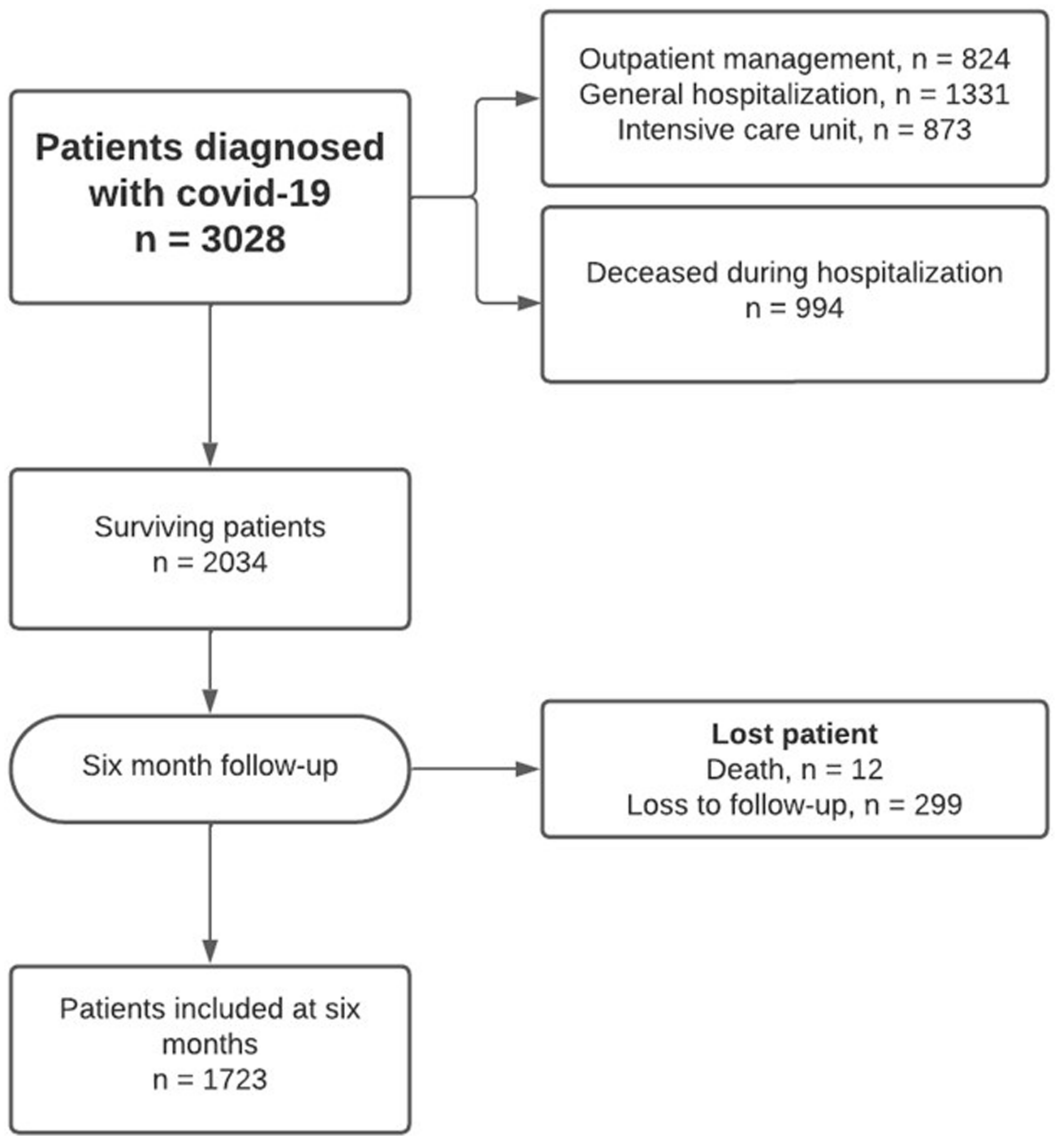
Flowchart of included patients in the analysis.

**Figure 2:**
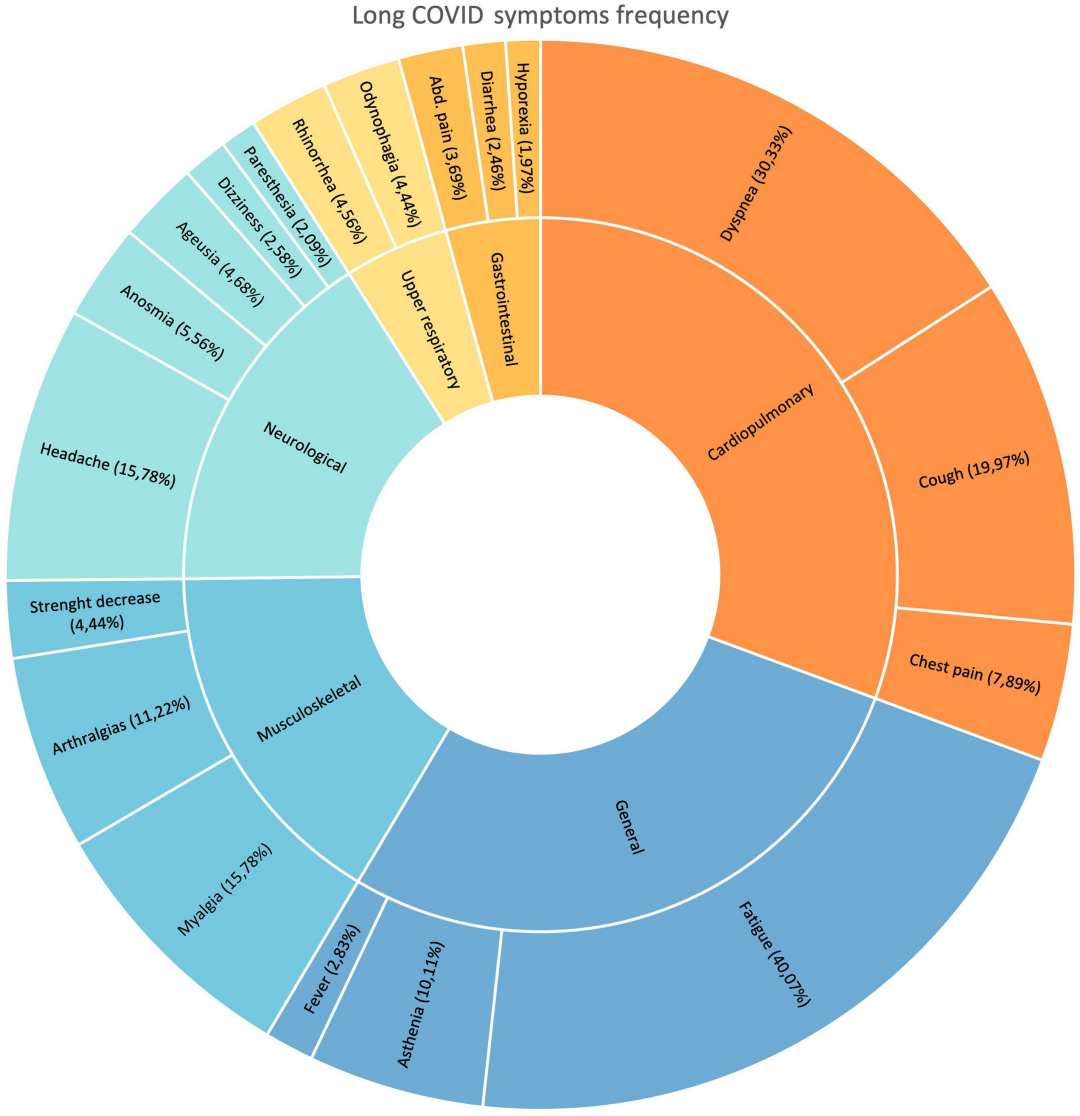
Sunburst symptom frequency in patients with long COVID. Some patients exhibited more than two symptoms.

We included a total of 1,723 participants (Figure 1), of which 55.02% were female. The median age of the participants was 53 years, and the prevalence of Long Covid was found to be 47.07%. The predominant symptoms were of a general (chills, asthenia, fatigue, and fever) and cardiopulmonary (dyspnea, chest pain, and cough) nature. Fatigue was the most common symptom (40.07%), followed by dyspnea (30.33%) and cough (19.98%). Additionally, headache (15.78%) and myalgias (15.28%) featured prominently among the reported symptoms in the Long Covid population. Figure 2 illustrates other symptoms reported by participants in our study.

### 3.1 Population characteristics

In the Long COVID group (LCG), the median age was higher at 55 (IQR 37–66) years, while those in the No Long COVID group (NLC) had a median age of 51 (IQR 33–63) years. LCG exhibited a higher average BMI compared with the NLC (27 vs. 26, *p* = 0.028) (Table 1).

**Table 1:** Population characteristics.

	No long covid (NLC)	Long Covid (LCG)	Total	*p* value
	912 (52.93%)	811 (47.07%)	1,723 (100%)	
Age	51 (33–63)	55 (37–66)	53 (35–65)	<0.001
Gender				
Female	484 (53.07%)	464 (57.21%)	948 (55.02%)	0.084
Male	428 (46.93%)	347 (42.79%)	775 (44.98%)	
Body mass index	26 (24–29)	27 (24–30)	27 (24–30)	0.028
Smoking	124 (13.60%)	134 (16.52%)	258 (14.97%)	0.089
Current smoking	15 (12.10%)	6 (4.44%)	21 (8.11%)	0.024
Passive smoking	69 (7.57%)	66 (8.14%)	135 (7.84%)	0.659
Biomass exposure	65 (7.13%)	93 (11.47%)	158 (9.17%)	0.002
Alcohol consumption	131(14.36%)	137 (16.89%)	268 (15.55%)	0.424
Comorbidities at admission				
Hypertension	245 (26.86%)	264 (32.55%)	509 (29.54%)	0.010
Ischemic heart disease	30 (3.29%)	32 (3.95%)	62 (3.60%)	0.465
Heart Failure	14 (1.54%)	27 (3.33%)	41 (2.38%)	0.015
Chronic obstructive pulmonary disease	19 (2.08%)	38 (4.69%)	57 (3.31%)	0.003
Diabetes	122 (13.38%)	124 (15.29%)	246 (14.28%)	0.257
Obesity	183 (20.07%)	209 (25.77%)	392 (22.75%)	0.005
Dyslipidemia	111 (12.17%)	137 (16.89%)	248 (14.39%)	0.005
Rheumatic disease	16 (1.75%)	35 (4.32%)	51 (2.96%)	0.002
Cáncer	44 (4.82%)	31 (3.82%)	75 (4.35%)	0.309
Neurological disease	30 (3.29%)	29 (3.58%)	59 (3.42%)	0.744
Charlson index > = 3	220 (24.12%)	237 (29.22%)	457 (26,52%)	0.018

Chi^2^ test for categorical variables and Mann–Whitney U for continuous variables.

Around 9.17% of the total population reported exposure to biomass combustion (LCG: 11.47% vs. NLC: 7.13%, *p* = 0.002). Regarding alcohol consumption and tobacco use, there were no significant differences observed between both groups.

Comorbidities among patients upon admission to the emergency department revealed that metabolic diseases, including diabetes and obesity, were among the most prevalent. Additionally, cardiovascular diseases, such as a history of heart attacks, hypertension, and heart failure, were notably prevalent, with higher rates observed in LCG (Table 1).

### 3.2 Hospitalization characteristics

Among the total population, 36.85% received outpatient care, whereas 63.14% necessitated hospital management (49.3% were managed in general hospital wards, and 13.64% required Intensive Care Unit [ICU] admissions). The median length of hospital stay was longer in the LCG (9 vs. 7 days, *p* < 0.001) (Table 2).

**Table 2:** Hospitalization characteristics.

	No long COVID (NLC)	Long COVID (LCG)	Total	*p* value
	912 (52.93%)	811 (47.07%)	1,723 (100%)	
Outpatient	386 (42.32%)	249 (30.70%)	635 (36.85%)	<0.001
General hospitalization	446 (48.90%)	409 (50.43%)	855 (49.62%)	<0.001
ICU hospitalization	80 (8.77%)	153 (18.87%)	233 (13.52%)	<0.001
Length of hospital stay	7 (5–11)	9 (6–15)	8 (5–13)	<0.001
Length of ICU stay	9 (5–21)	15 (8–28)	13 (6–25)	0.005
Invasive mechanical ventilation requirement	32 (3.51%)	91 (11.22%)	123 (7.14%)	<0.001
Duration of Invasive mechanical ventilation	17 (10–28)	18 (10–32)	18 (10–30)	0.506
Tracheostomy requirement	16 (3.01%)	44 (7.83%)	60 (5.49%)	<0.001
Pronation requirement	78 (14.69%)	154 (27.40%)	232 (21.23%)	<0.001

Chi^2^ test for categorical variables and Mann–Whitney U for continuous variables.

Furthermore, it was observed that 7.14% of the total population also needed invasive mechanical ventilation. Among those with LC, there was a higher proportion of tracheostomies (7.83% vs. 3.01%, *p* < 0.001) and a greater demand for prone positioning during their hospitalization (27.40% vs. 14.69%, *p* < 0.001).

### 3.3 Binomial regression

In the multivariate analysis the variables associated with presence of LC were age, sex, the presence of comorbidities such as high blood pressure, rheumatologic diseases, diseases related to respiratory system, and metabolic diseases, and different aspects of hospitalization and medical interventions, such as the type of hospital stay, ventilation support, and tracheostomy procedures (Table 3).

**Table 3:** Binomial regression model.

Variable	Unadjusted			Multivariate		
	RR	95% CI	*p*-Value	RR	95% CI	*p*-value
Age	1.005	(1.002–1.008)	<0.001	0.999	(0.996–1.002)	0.606
Gender(Female)	1.093	(0.987–1.210)	0.086	1.148	(1.038–1.268)	0.007
Body mass index	1.008	(0.998–0.018)	0.128			
Smoking	1.124	(0.987–1.280)	0.089			
Current smoking	0.527	(0.265–1.047)	0.024			
Biomass exposure	1.283	(1.114–1.477)	0.002			
Alcohol consumption	1.104	(0.970–1.256)	0.148			
Hypertension	1.151	(1.037–1.278)	0.010			
Heart failure	1.413	(1.127–1.772)	0.015			
Chronic obstructive pulmonary disease	1.437	(1.187–1.739)	0.003	1.340	(1.104–1.626)	0.003
Obesity	1.179	(1.056–1.316)	0.005			
Dyslipidemia	1.209	(1.067–1.370)	0.005			
Rheumatic disease	1.479	(1.220–1.793)	0.002	1.260	(1.055–1.504)	0.011
Charlson index	1.040	(1.018–1.064)	<0.001			
Charlson index ≥ 3	1.144	(1–028–1-273)	0.014			
General hospitalization	1.033	(0.934–1.142)	0.527	1.247	(1.090–1.427)	0.001
ICU hospitalization	1.487	(1–333-1.658)	<0.001	1.490	(1.221–1.818)	<0.001
Invasive mechanical ventilation	1.644	(1.461–1.850)	<0.001	1.278	(1.050–1.555)	0.014
Tracheostomy	1.462	(1.241–1.723)	<0.001			
Pronation	1.401	(1.248–1.572)	<0.001			

**Figure 3:**
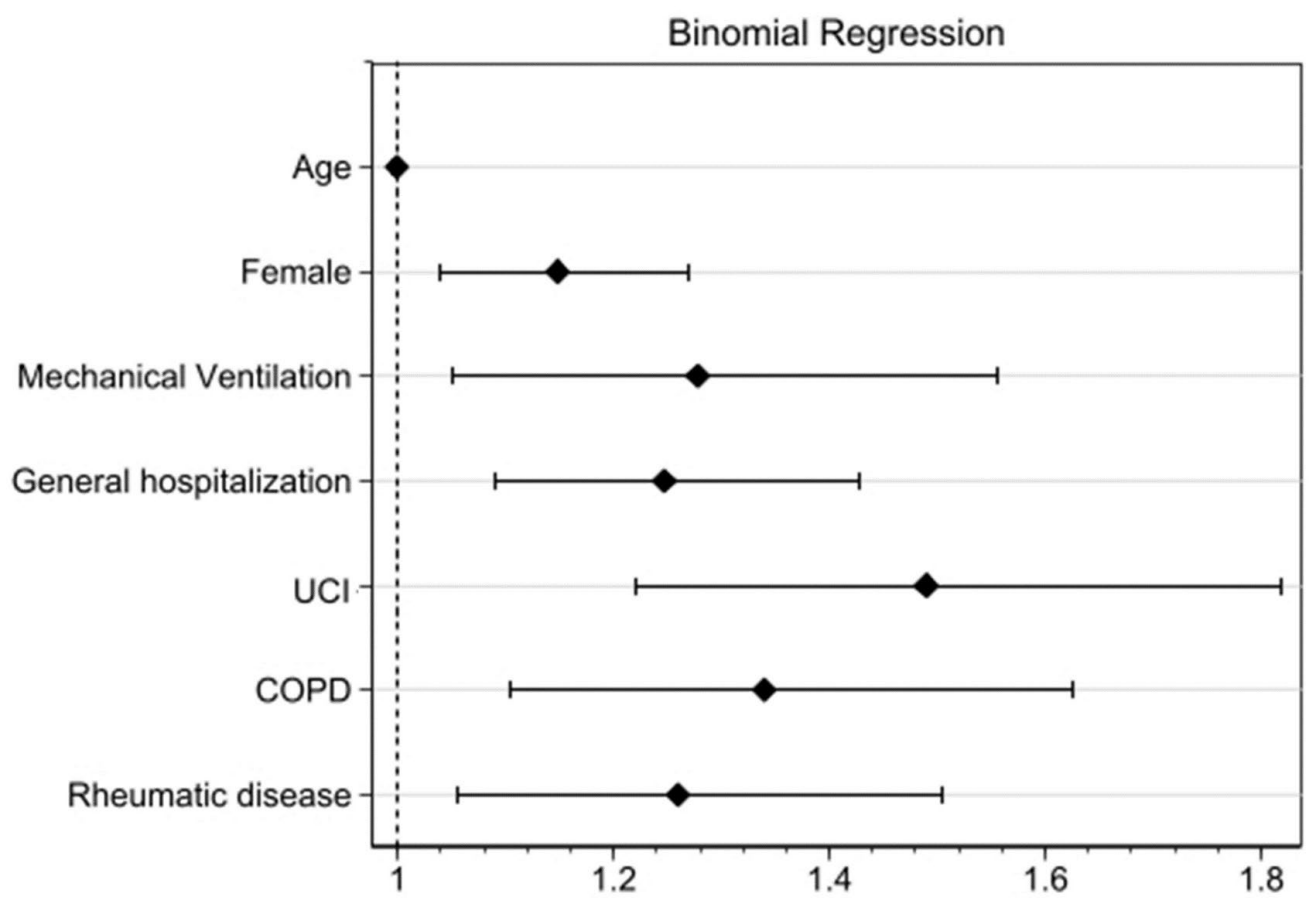
Binomial regression of variables associated with development of long COVID.

**Figure 4:**
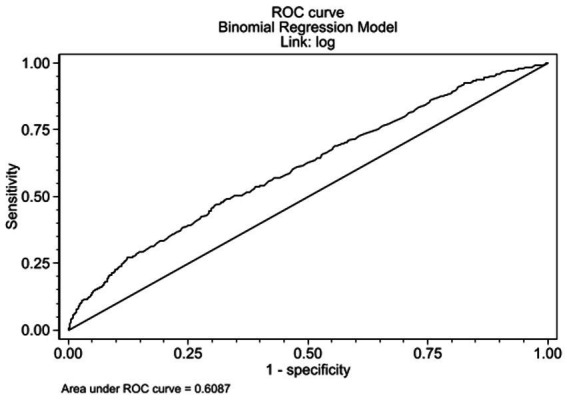
ROC curve binomial regression model.

In the multivariate binomial regression model, Age demonstrated no significant association with LC (RR 0.999 [95% CI 0.996–1.002]; *p* = 0.606). However, female sex (RR 1.148 [95% CI 1.038–1.268]; *p* = 0.007), requirement of mechanical ventilation (RR 1.278 [95% CI 1.050–1.555]; *p* = 0.014), presence of Chronic Obstructive Pulmonary Disease (COPD) (RR 1.340 [95% CI 1.104–1.626]; *p* = 0.003), presence of Rheumatic Disease (RR 1.259 [95% CI 1.055–1.504]; *p* = 0.011) and the Hospitalization Type: General Hospitalization (RR 1.247 [95% CI 1.090–1.427]; *p* = 0.001) and ICU Hospitalization (RR 1.490 [95% CI 1.221–1.818]; *p* < 0.001) were significantly associated with the persistence of symptoms at the sixth month of follow-up. This model showed an area under the curve of 0.608 (Figures 3, 4).

## 4 Discussion

Despite the current decline in COVID-19 incidence, its repercussions persist within our population, attributable to the lingering symptoms that continue to impact the quality of life for those affected. Our study found a Long COVID prevalence of 47.07% in this population, with the most prevalent symptoms being of a general and cardiopulmonary nature (fatigue, dyspnea, and cough). Additionally, individuals with Long COVID exhibited a slightly higher average BMI and were more likely to have exposure to biomass combustion and comorbidities.

Similar findings were reported in a cohort in Russia, where at the 6-month mark, 50% of adults presented PCC (18). However, these findings present a contrast to those observed in the Mexican Cohort, where a prevalence of 83.3% was documented at the 6-month mark. Nevertheless, it is imperative to consider that this cohort encompassed individuals with severe COVID-19, and the most frequently reported symptoms were respiratory (use of supplemental oxygen and dyspnea), which were analyzed at 90 days of discharge (12). Additionally, another study conducted in Iran reported that 62.3% of the patients evaluated at the 3-month mark exhibited symptoms consistent with Long COVID (LC). The most frequently reported manifestations included fatigue, exercise intolerance, walking intolerance, muscle pain, and shortness of breath (19). The disparities in prevalence rates may be attributed to the distinct patient populations under consideration and the varying timelines for symptom assessment. The higher prevalence of Long COVID observed in females is consistent with previous findings, which have suggested a sex-based disparity in the development of this condition (20, 21). The exact mechanisms underlying this disparity are not fully understood, but potential explanations include hormonal differences, variations in the immune system, and genetic factors (22). This association was further substantiated by the findings in the Iranian and Russian cohort, where female sex emerged as an identified factor linked to the development of Long COVID syndrome (18, 19).

The association between Long COVID and hospitalization is well-established (23). This study further revealed that individuals with Long COVID were more likely to have required hospitalization and mechanical ventilation during the acute infection. This finding underscores the severity of respiratory distress in the Long COVID population (24). COPD has been associated with severe presentations of COVID-19 (24), with some cases attributing the severity to the pre-existing lung damage characteristic of COPD (25). However, the association between COPD and LC has received relatively less attention in the research landscape. This knowledge gap may be due, in part, to the challenge of distinguishing Long COVID symptoms from those of COPD, potentially leading to symptom overlap (26).

Most of the studies related to this subject have primarily centered on hospitalized patients. However, the higher prevalence of LC is consistently noted in this subgroup, this could be linked to the severity of illness (23, 27). A prospective cohort study conducted in Northwest Spain yielded findings akin to our study; among the various comorbidities considered, only COPD exhibited a statistically significant association with a higher prevalence of persisting symptoms 6 months after COVID-19 (23). In contrast to the previous study, our research identified another associated comorbidity, the presence of rheumatic diseases.

In our study, LC was found to be more prevalent among patients with inflammatory rheumatic diseases compared to healthy controls, which is consistent with the results of Boekel et al. (28). The emerging body of evidence underscores the potential predisposition of rheumatic patients to LC, attributed to alterations in immune regulatory responses (29). Also, it had been found that up to 45 percent of individuals living with rheumatic diseases, encompassing conditions such as rheumatoid arthritis and other chronic autoimmune disorders characterized by inflammation, exhibit persistent symptoms associated with Long COVID even 28 days after the acute SARS-CoV-2 infection (28).

For our cohort, the binomial regression analysis identified several factors associated with an increased risk of Long COVID, including age, female sex, hospitalization, invasive mechanical ventilation, and the presence of comorbidities such as COPD and rheumatic disease. These findings suggest that individuals with these characteristics may be at a higher risk of developing Long COVID and should be closely monitored.

Understanding the prevalence and identifying associated factors of LC allows for the characterization of the population at risk of developing this condition. This, in turn, propels the development of tools for early and timely diagnosis of the pathology. Additionally, it provides a logical explanation and reassurance regarding the symptoms for patients experiencing long COVID.

## 5 Limitations

This study has several limitations. Firstly, it was conducted in a single country, potentially limiting the generalizability of the findings. Nevertheless, a key strength of our study lies in the substantial number of patients from whom we obtained data. They were systematically followed up, and surveys were administered in a structured manner. Secondly, the study relied on self-reported data, a condition that is still poorly defined and susceptible to recall bias. Thirdly, data gathering at cohort entry occurred at the onset of the pandemic, leading to the omission of several variables of interest, such as vaccines. Despite this, the number of variables included was deemed sufficient for the study. It is crucial to emphasize that patient follow-up and survey administration were conducted in a structured manner.

## 6 Future research directions

Future research should focus on addressing the limitations of this study. Larger, multicenter studies with longitudinal follow-up are needed to confirm the findings of this study and to assess the long-term outcomes of individuals with Long COVID. Additionally, future research should focus on elucidating the underlying mechanisms of Long COVID and developing effective prevention and treatment strategies.

## 7 Conclusion

This study revealed a high prevalence of Long COVID, impacting nearly 50% of individuals recovering from COVID-19. General and Cardiopulmonary symptoms were the most frequently reported. LC was found to be more prevalent among females and those with COPD and rheumatologic disease, along with other underlying medical conditions. These results emphasize the urgency of conducting additional research to elucidate the etiology of LC and to formulate efficacious therapeutic strategies. Healthcare providers must remain vigilant regarding the substantial occurrence of LC and its associated risk factors. Additionally, a thorough evaluation of patients for LC symptoms is crucial, enabling the development of tailored management strategies.

## Data availability statement

The raw data supporting the conclusions of this article will be made available by the authors, without undue reservation.

## Ethics statement

The studies involving humans were approved by Fundación Oftalmologica de Santander Ethics and Research Committee. The studies were conducted in accordance with the local legislation and institutional requirements. The participants provided their written informed consent to participate in this study.

## Author contributions

MM-A: Conceptualization, Formal analysis, Methodology, Writing – original draft. NP: Writing – review & editing. JC-D: Formal analysis, Methodology, Writing – original draft. AL-M: Formal analysis, Investigation, Writing – review & editing. CC-R: Investigation, Writing – review & editing. SV-F: Writing – review & editing. PC-L: Formal analysis, Writing – review & editing.
